# An End to Endless Forms: Epistasis, Phenotype Distribution Bias, and Nonuniform Evolution

**DOI:** 10.1371/journal.pcbi.1000202

**Published:** 2008-10-24

**Authors:** Elhanan Borenstein, David C. Krakauer

**Affiliations:** 1 Department of Biological Sciences, Stanford University, Stanford, California, United States of America; 2 Santa Fe Institute, Santa Fe, New Mexico, United States of America; University of Washington, United States of America

## Abstract

Studies of the evolution of development characterize the way in which gene regulatory dynamics during ontogeny constructs and channels phenotypic variation. These studies have identified a number of evolutionary regularities: (1) phenotypes occupy only a small subspace of possible phenotypes, (2) the influence of mutation is not uniform and is often canalized, and (3) a great deal of morphological variation evolved early in the history of multicellular life. An important implication of these studies is that diversity is largely the outcome of the evolution of gene regulation rather than the emergence of new, structural genes. Using a simple model that considers a generic property of developmental maps—the interaction between multiple genetic elements and the nonlinearity of gene interaction in shaping phenotypic traits—we are able to recover many of these empirical regularities. We show that visible phenotypes represent only a small fraction of possibilities. Epistasis ensures that phenotypes are highly clustered in morphospace and that the most frequent phenotypes are the most similar. We perform phylogenetic analyses on an evolving, developmental model and find that species become more alike through time, whereas higher-level grades have a tendency to diverge. Ancestral phenotypes, produced by early developmental programs with a low level of gene interaction, are found to span a significantly greater volume of the total phenotypic space than derived taxa. We suggest that early and late evolution have a different character that we classify into micro- and macroevolutionary configurations. These findings complement the view of development as a key component in the production of endless forms and highlight the crucial role of development in constraining biotic diversity and evolutionary trajectories.

## Author Summary

At the very end of his *On the Origin of Species*, Charles Darwin wrote, “from so simple a beginning endless forms most beautiful and most wonderful have been, and are being, evolved.” Nature truly displays a bewildering variety of shapes and forms. Yet, with all its magnificence, this diversity still represents only a tiny fraction of the endless “space” of possibilities; research on the evolution of development has revealed that observed common morphologies and body plans (or, more generally, phenotypes) occupy only small, dense patches in the abstract phenotypic space. In this paper, we introduce a simple model of evolving gene regulation and show that these empirically identified patterns can be attributed, at least in part, to interaction between genes (epistasis) in the developmental network. Our model further predicts that early developmental programs with low levels of interaction would span most of the variation found in extant species. The theory presented in our paper complements the view of development as a key component in the production of endless forms and highlights the crucial role of development in constraining (as well as generating) biotic diversity.

## Introduction

The tremendous diversity of shapes and forms observed in nature is truly remarkable and yet it represents only a small fraction of the ‘space’ of the possible. One reason for this is that the space of possible genotypes has been incompletely sampled over the course of the history of life on earth. If we consider the astronomical volume of the genotypic space, then the set of all DNA strands that were ever produced during earth history constitute a tiny fraction of the total sequence space. Moreover, the genotypes that have existed are the result of an evolutionary process—descent with modification from a common ancestor—which is a locally-delimited generative process. Phenotypic diversity is further constrained by another process, one intrinsic to the manufacture of adaptive varieties, the developmental mechanisms that determine the mapping of genotypes into phenotypes.

Development induces a non-linear and highly degenerate mapping from gene-space to phenotype space, whereby many genotypes produce similar (or identical) phenotypes, and concomitantly, ensuring that there are many phenotypes that cannot be generated by any genotype. This arises from both neutral genetic properties of the developmental dynamic, and from the evolution of robustness mechanisms which seek to preserve functional phenotypes in the face of environmental and genetic variation [1],[2]. Degeneracy has the effect of hiding genotypes from the selective process and rendering a large portion of potential phenotypes inaccessible. This is an architectural constraint that limits available variation and adaptive capacity, with potentially dramatic effects on the trajectory of the evolutionary process. Whereas evolutionary search over the space of frequently generated phenotypes is in strict accordance with neo-darwinian theory (population genetics for example), the sparse distribution of the phenotypic space has implications for large scale patterns of evolutionary change, and this can only be appreciated through the introduction into the evolutionary dynamic of a suitable model of development.

Developmental mappings are generally extremely complex. This complexity derives from a combination of hierarchical regulation, multi-gene control, epistasis, and pleiotropy. A large body of work examines the statistical and dynamical properties of developmental maps in simple systems, focusing on neutrality and neutral networks of RNA [3]–[5] and on gene regulatory networks in multicellular development [6]–[8]. These studies have generated interest among paleontologists inquiring into the origin and diversification of body plans [9]–[11] and have lead to the suggestion that morphological variation is extensive early in the history of multicellular life [9],[12], that phenotypes are sparsely distributed in the space of ‘potential’ phenotypes [13], and that diversity is better predicted by variation in the structure of gene regulation networks than variation in the presence and absence of structural genes [14].

Here, we consider a very generic property of complex developmental maps—the interaction between multiple genetic elements and the non-linearity of gene interaction—in shaping various aspects of a phenotype. On the mechanistic genetic level, this is usually referred to as *epistasis* and *pleiotropy*, but the same generic constraint principle might also apply to many other biological mappings, ranging from the physical interactions between amino acids in the production of protein structures, to the interactions between tissues and their effects on gross morphology. We wish to show that a basic geometric property of development provides a null model able to account for the bias and nonuniformity of phenotype distributions.

The model is constructed as a generic representation, capturing the way multiple genetic inputs combinatorially interact to influence multiple phenotypic traits, and does not assume selection. One natural interpretation is that of cis-regulatory architecture and gene interaction [14]–[16]. For convenience, we use terms related to this interpretation throughout the paper. We use the model to examine a number of statistical regularities of the developmental map that it induces. In particular, we derive the fraction of visible phenotypes generated during development and the dependence of this fraction on the level of interaction between genetic elements. We characterize the distances among visible and frequently occurring phenotypes and the influence of development on phylogenetic relationships. We demonstrate that many of the empirical, developmental and paleontological regularities summarized above can be recovered using this null model.

## Models

### Basic Model

Genotypes and phenotypes are represented as binary vectors of lengths *r* and *k*. Generally speaking, genotypes represent the presence/absence of *r* genetic elements (e.g., genes, alleles, etc.), and phenotypes represent the presence/absence of *k* phenotypic traits. An interpretation in terms of cis-regulatory dynamics posits that genotypes represent the expression pattern of a set of *r* transcription factors (TFs) and that phenotypes denote the expression pattern of *k* target genes regulated by these TFs. In this sense, genotypes and phenotypes in our model may be viewed as representing certain aspects of the cell transcriptional state. In the following, we refer to *r* as the regulatory dimension and to *k* as the phenotypic dimension.

**Figure 1:**
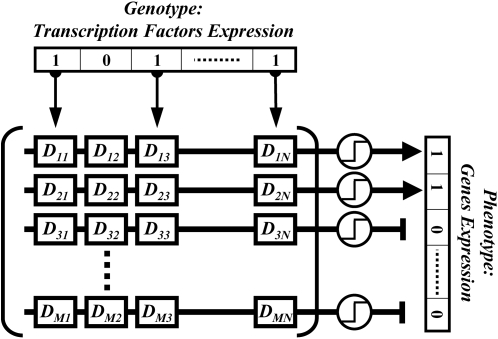
An illustration of the developmental model. The *r* transcription factors bind to the promoters of *k* structural genes with affinities given by *D_ij_*. If the net activation to a promoter exceeds a threshold value (illustrated as a step function) the gene is expressed. The phenotype is described by the distribution of gene expression. This regulatory architecture corresponds to the single layered plan - See also our analysis of a generalized, multilayered, model.

A developmental plan maps genotypes to phenotypes. We define a developmental plan as a matrix, *D*, of size *k*×*r*. Each entry in this matrix is either +1 or −1 with equal probability (using real numbers drawn from a uniform or Gaussian distribution with mean 0 does not qualitatively change the results presented in this paper). Given a genotype, 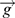, the phenotype to which it maps is calculated by 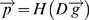, where *H* denotes the heaviside function (i.e., the unit step function centered at zero). In the regulatory interpretation, *D_ij_* describes properties of the binding site for transcription factor *j* in the promoter of gene *i* (Figure 1, and see [15],[17]). The heaviside function can alternatively represent a switching mechanism, producing a signal only if inputs exceeds a threshold value.

In the analysis presented throughout the paper, we enumerated *all* 2*^r^* possible genotypes and used a fixed, randomly generated developmental plan *D* to map these genotype onto the corresponding 2*^r^* phenotypes. To obtain large-scale statistics for the distribution of visible phenotypes and their relationships, we repeated this process, using numerous developmental plans. The distinction between a ‘structural’ part of the genome (which is allowed to vary) and a developmental part (that remains fixed), is motivated by our attempt to explore the implications of a given plan on the distribution of phenotypes, and by the suggestion that developmental plans form a mechanical basis for phylogenetic grades [7] (see also Discussion).

### Multilayered Models

Previous studies on the evolution of development, have considered a dynamical *recurrent* model of gene regulation. In these models, the resulting ‘phenotype’ (or pattern of gene expression) is fed back into the regulatory plan, until the system reaches steady state [15],[17]. These models aim to capture multilayered plans, where the output of one layer forms the input to the next (these recurrent models are a simple case where the same plan is applied to each layer). By contrast, the model described above employs a single layer architecture. To examine the effect of such multilayered plans we extend our model by allowing it to include multiple regulatory layers. Formally, we define 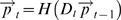, where *t* denotes the regulatory layer number and the initial phenotype, 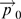, corresponds to a given genotype 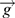 (as described in our original model). We iterate this model repeatedly, starting with a collection of all possible 2*^r^* genotypes, and record the phenotype distribution at each layer.

### Partially Connected Models

Our basic model assumes a ‘fully connected’ regulatory plan, wherein all entries in the developmental matrix *D* are nonzero: every gene in the genotype affects every element in the phenotypic vector. Previous models have considered sparser interaction plans and examined the effect of varying the density of the regulatory interactions. Specifically, the density of the regulatory plan has been shown to have important effects on the consequences of gene duplications [18], epigenetic stability [15], the evolution of canalization [17], and robustness [19]. We therefore further extend our model by introducing an additional parameter, *c*, which denotes the density of the matrix *D* (i.e., the probability for each entry in the matrix to be attributed with a nonzero value; *c* = 1 corresponds to a fully connected plan), and examine its effect on the distribution of phenotypes.

## Results

### Potential and Visible Phenotypes

**Figure 2:**
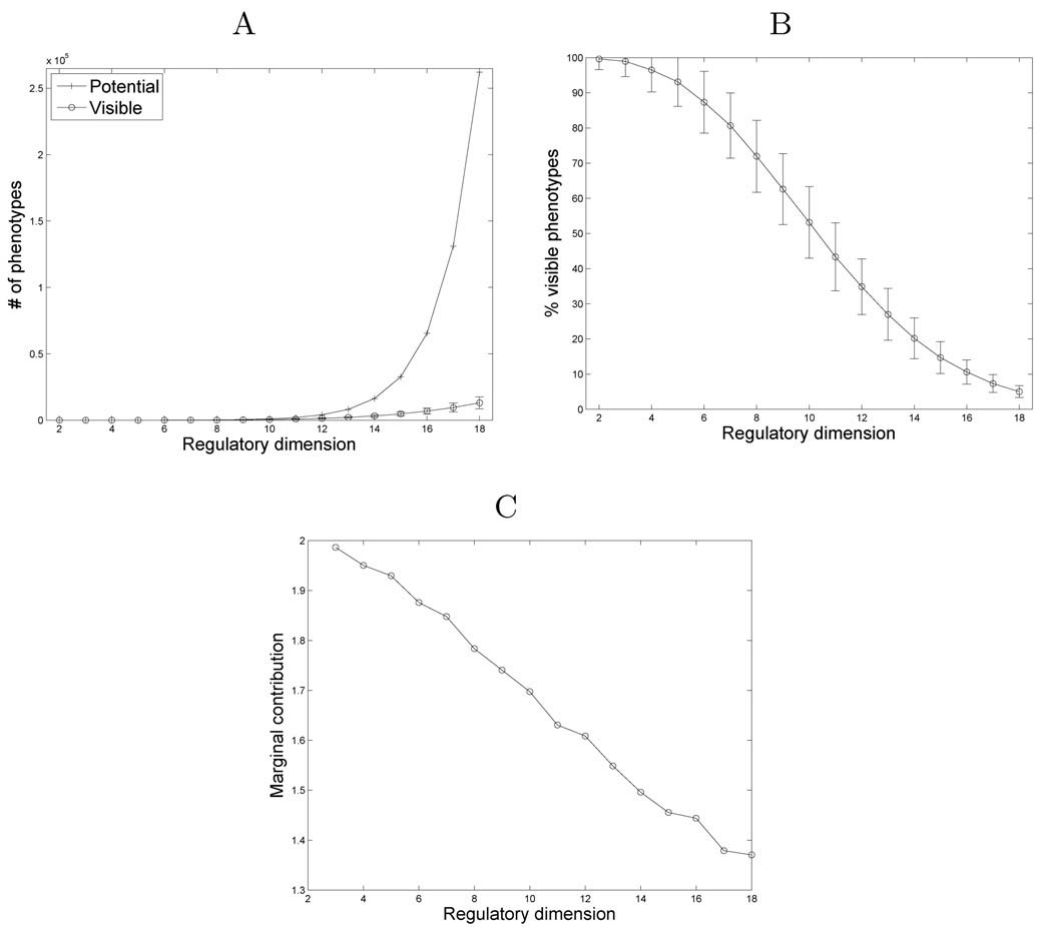
Potential and visible phenotypes as a function of the regulatory dimension, *r*. The phenotypic dimension is set to *k* = 18. All curves represent the average of 1,000 different developmental matrices. (A) The number of potential phenotypes (2*^r^*) and the number of distinct visible phenotypes as a function of the regulatory dimension. (B) The percentage of visible phenotypes out of the potential phenotypes, corresponding to a sigmoidal function. (C) The marginal contribution of each genetic element to the increase in the number of visible phenotypes. Formally, if *V*(*r*) denotes the number of visible phenotypes as a function of *r*, then the marginal contribution is defined as *V*(*r*)/*V*(*r*−1), and is evidently linear (with slope of −0.044; least squares regression).

Consider a developmental model with regulatory dimension *r* and phenotypic dimension *k*. There are 2*^r^* genotypes which could produce a maximum of 2*^r^* phenotypes. However, the developmental plan maps several genotypes into the same phenotype (giving rise to degeneracy), and consequently generates a much smaller number of distinct phenotypes. We refer to the set of phenotypes produced by a given developmental plan as *visible phenotypes*, and examine the number of visible phenotypes and the number of potential phenotypes as a function of *r* (Figure 2A). We find that while the *number* of visible phenotypes increases with the regulatory dimension, *r*, their fraction, out of the number of potential phenotypes, rapidly declines, with around 5% of the potential phenotypes remaining visible (Figure 2B) when *r* = *k*. In other words, the expansion of the genotypic space, which also promotes an expansion in the number of possible genotypic configurations, also brings about an increased canalization, masking the expansion in the number of new visible phenotypes. This is further exemplified by the marginal contribution of each genetic element (e.g., each transcription factor), measured as the relative increase in the number of visible phenotypes obtained by adding a new genetic element. This declines from about two-fold for the first few elements, to less than 1.4 as *r* reaches *k* (Figure 2C). As per the mathematical analysis below, this can be attributed to the effect of an unbalanced sample of *D* entries and the multiplicative effect of the nonuniform distribution of each element in the phentype. Interestingly, the function describing the fraction of visible phenotypes (Figure 2B) is sigmoidal, with the greatest change in the fraction of visible phenotypes occurring at regulatory dimensions on the order of half the phenotypic dimension. Thus for smaller regulatory circuits, a large fraction of potential phenotypes remain visible, whereas for larger regulatory circuits, the greater fraction of the phenotypic space is hidden and inaccessible to selection and evolutionary transformation.

### Localization of the Visible Phenotypic Subspace

**Figure 3:**
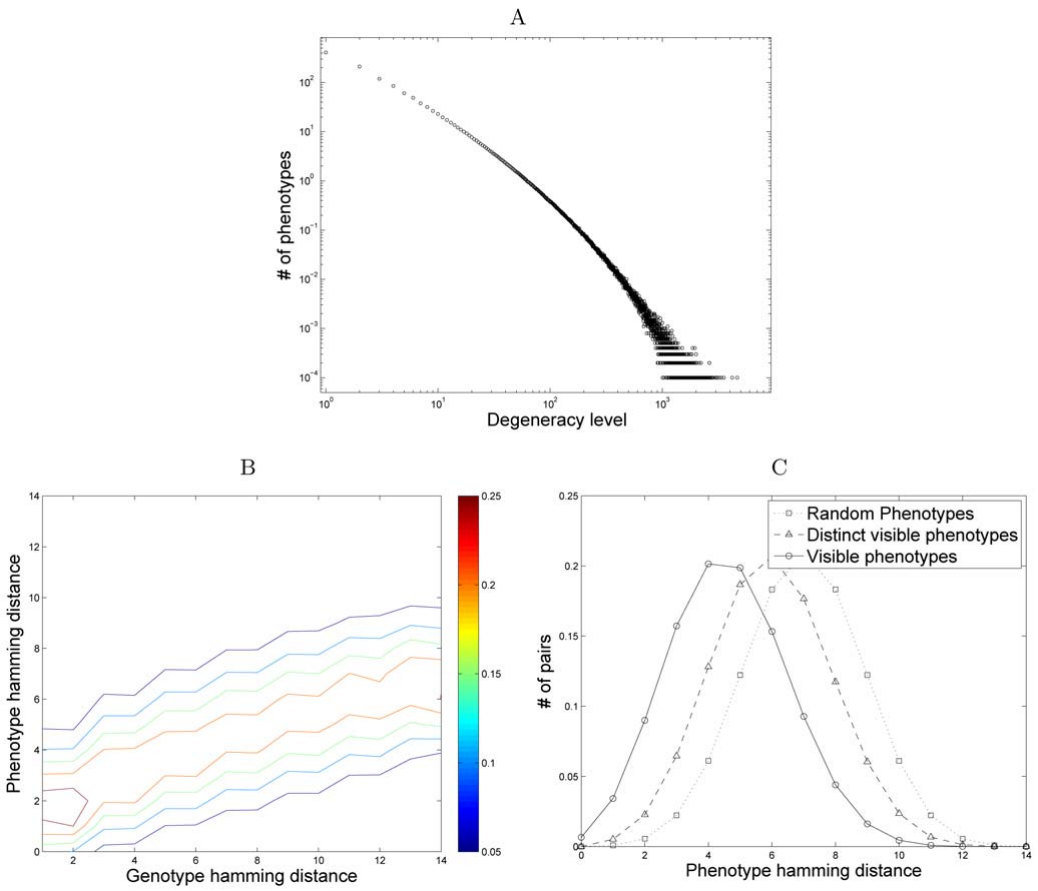
Localization of the visible phenotypic subspace. (A) A loglog plot of the distribution of degeneracy levels among visible phenotypes. Each point denotes the expected number of distinct phenotypes with a certain degeneracy level for a given developmental plan and is an average over 10,000 different plans. Note that the point associated with degeneracy level 0 (i.e., hidden phenotypes) is not included. These developmental plans frequently give rise to phenotypes with degeneracy levels higher than 10^3^, and in rare cases, higher than 10^3.5^. Given that the total number of genotypes is 2^14^ a single phenotype can be produced by 6%–20% genotypes. (B) A contour plot of the gain function induced by a given developmental plan (all developmental plans produce qualitatively similar results). The gain function, *gain*(*d_g_*,*d_p_*), denotes the probability that the Hamming distance between two phenotypes is *d_p_*, given that the distance between the two genotypes that produced them is *d_g_*. (C) The distribution of pairwise phenotypic Hamming distances among randomly selected phenotypes (not produced by a developmental plan), distinct visible phenotypes (considering every visible phenotype only once, regardless of frequency), and visible phenotypes including all occurrences of each phenotype. The pairwise Hamming distances between randomly selected phenotypes follows a binomial distribution, with mean distance 7 (for phenotypes of length 14). Distinct visible phenotypes are closer to one another, with the mean distance 5.976. When weighting by the frequency of the visible phenotypes, the distance is reduced, with a mean distance 4.607.

Having established that large regulatory networks lead to a small number of visible phenotypes, we turn to the statistical characteristics of the visible, phenotypic subspace. We focus on developmental plans for which *r* = *k* (e.g., the number of TFs matches the number of target genes). As demonstrated above, these developmental plans produce the most restricted set of visible phenotypes. Unless otherwise indicated, we set *r* = *k* = 14 to allow for the complete enumeration of all genotypes. We consider the distribution of frequency levels among the visible phenotypes. We calculate for each phenotype *j*, a *degeneracy level*, *n_j_*, denoting the number of different genotypes that produce it (visible phenotypes correspond to those phenotypes for which *n_j_*>0). The distribution of degeneracy levels fits a generalized power-law distribution (Figure 3A), implying that there are a few very common (frequent) phenotypes and many rare ones. These findings replicate those on the highly nonuniform frequencies of folding geometries in the RNA secondary structure genotype/phenotype map [20].

Are the visible phenotypes uniformly distributed across the phenotypic space or clumped in a nonuniform, subspace of closely related phenotypes? To answer this question, we compute the gain function of a given developmental plan. This function describes the distribution of Hamming distances among phenotypes (using the average pairwise dissimilarity [21]) whose origins are genotypes a certain Hamming distance apart. In other words, the gain function measures how the magnitude of a perturbation in the genotype space maps onto perturbations in the phenotype space. The resulting gain function suggests that the developmental plan induces a significant degree of canalization; a large perturbation in the genotype space (measured as the Hamming distance between the original and perturbed genotypes) produces, on average, a significantly smaller perturbation in the phenotype space (Figure 3B). This canalization compresses the image of the genotypes in the phenotype space, and promotes a patchy subspace. This property of regulatory networks has been adduced as evidence for the incremental evolution of developmental robustness [19].

To further characterize the patchiness of the visible, phenotypic subspace, we calculate the distribution of pairwise Hamming distances between visible phenotypes, this time not conditioning on the distance between their genotypes (Figure 3C). Here we plot the frequency distributions of pair-wise Hamming distances under three different conditions: (1) randomly drawn phenotypes from the space of potential phenotypes, (2) randomly drawn phenotypes from the set of distinct, visible, phenotypes, and (3) randomly drawn phenotypes from the set of visible phenotypes including all occurrences of each phenotype. In other words, each distinct, visible phenotype is sampled with a probability proportional to its frequency. We find that the condition involving the visible phenotypes, tends to generate phenotypes more similar than expected by chance (random phenotype distribution). Moreover, when controlling for frequency (the degeneracy level) of the visible phenotypes, we find that the distribution is further skewed toward smaller Hamming distances. This suggests that the most frequent phenotypes span a smaller subspace than the total visible phenotypes, and are located towards the center of the visible phenotype set.

**Figure 4:**
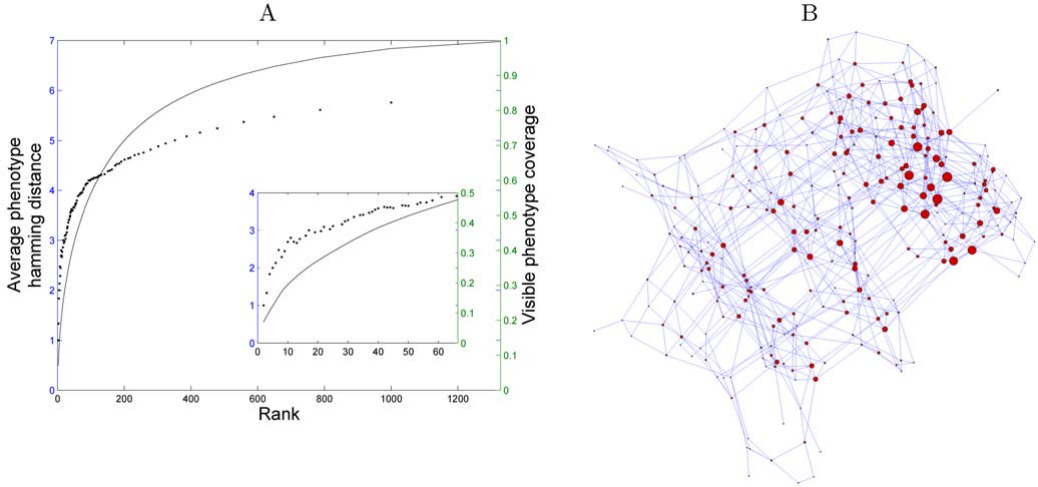
The average distance between the the most frequent phenotypes and the patchiness of the visible phenotypic subspace. (A) The average Hamming distance among visible phenotypes as a function of their frequency (dots). Visible phenotypes are ranked according to their frequency level. For each rank, we calculate the average Hamming distance between all visible phenotypes with this or higher rank. The most abundant phenotypes are very similar. This similarity decreases as less frequent phenotypes are included in the analysis. We also calculate which fraction of all visible phenotypes are included in these phenotypes (solid line). The inset shows a zoom of the same plot, focusing only on the top 5% most frequent phenotypes. The phenotypes that are included in this small fraction of the distinct visible phenotypes, are, on average, only 4 bits different, and still cover 50% of the phenotypes. (B) The one mutant neighbor network of the visible phenotypes. The size of the node is proportional to the logarithm of its frequency. In this plot, *r* = *k* = 12.

To examine this observation in greater detail we measure the average Hamming distance between visible phenotypes as a function of their frequency and represent them on a frequency-rank versus distance plot. The highest ranked phenotypes are presented as the lowest rank values. As shown in Figure 4A, the distance between the most frequent phenotypes is significantly smaller than the average distance (which in this case is ∼6), and increases as more visible phenotypes (with lower frequencies) are considered. Considering the case where all the visible phenotypes are included in this analysis, the average distance is still smaller than that expected by chance. We find that the top 5% most frequent phenotypes are very similar (average Hamming distance is smaller than 4) yet cover approximately 50% of all the visible phenotypes (4A inset). An additional illustration of this patchiness can be observed in Figure 4B, plotting the one mutant-neighbor network of all the visible phenotypes. Here we observe that the nodes that represent the most frequent phenotypes tend to be separated in most cases by a single edge.

### Statistical and Numerical Analysis

While an exact mathematical derivation for the nonuniform distribution of degeneracy levels and fraction of hidden phenotypes is hard to obtain, we consider an approximate, statistical approach in order to provide an intuition for their origin.

We first examine the expected statistical properties of a single trait element. Let *p_j_* denote the *j*th element of the phenotype. We consider complex, non linear mappings of the form: 
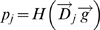
, where *H* denotes the heaviside function, 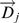 denotes the *j*th row of the developmental matrix *D*, and 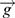 denotes a given genotype. The binary vector 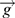, selects elements of 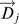 for summation. It follows that *Pr*(*p_j_* = 1) is the probability that the sum of the elements in a subset of 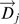 elements is greater than zero. Each element in 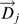 is either +1 or −1 with equal probability. Let *z_j_* denote the number of +1 elements in 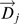. *z_j_* follows a binomial distribution *B*(*r*,0.5), where *r* is the regulatory dimension—the number of elements in 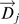. Let *s_g_* denote the number of nonzero elements in the genotype 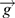. *Pr*(*p_j_* = 1) is the probability that a subset of size *s_g_* drawn without replacement from a set of *z_j_* number of +1 elements and *r*−*z_j_* number of −1 elements, contains more +1 elements than −1 elements. This probability is given by,
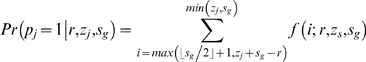
(1)where *f* denotes the hypergeometric probability mass function, 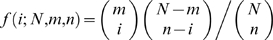. Furthermore, since in our model we consider all genotypes (all possible subsets of *r* choose *s_g_*) to be occupied by 1 or a 0 with equal probability, we can multiply our previous expression by the binomial probabilities for each element of the genome, to derive an average probability for each trait value :
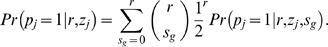
(2)


**Figure 5:**
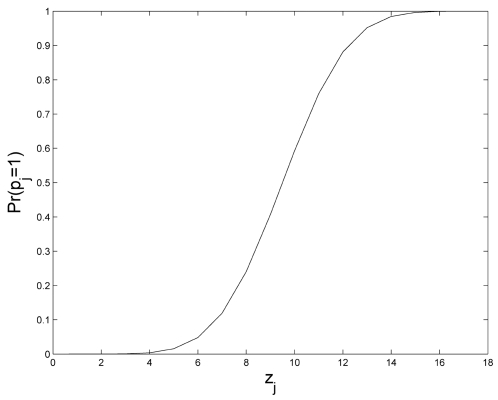
*Pr*(*p_j_* = 1), as a function of *s_j_*, the number of +1 elements in 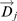. The total number of elements in 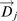, *r* = 18.

Figure 5 illustrates that *Pr*(*p_j_* = 1) is a sigmoidal function of *z_j_*. If *p_j_* had been determined by only one, randomly drawn, element of 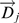, *Pr*(*p_j_* = 1) would be proportional (linearly) to the fraction of +1 elements in 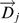. However, since *p_j_* is determined by a random subset, the consequences of a larger fraction of +1 elements is a combinatorial amplification. For example consider the case where 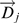 is comprised mostly of −1's with only very few +1 elements. A subset of 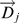 will typically have many more −1's than +1's, as there are exponentially many more ways to choose −1 elements than the +1 elements. We argue that this strong dependence of the phenotypic element on the number of +1 elements in the corresponding developmental matrix row is the source for the nonuniform distribution of degeneracy levels.

We next consider the entire phenotypic vector, rather than a single trait element *j*. Clearly, *Pr*(*p_j_* = 1) and *Pr*(*p_l_* = 1), the probabilities of producing 1 in the *j*th and *l*th elements of the phenotype, are not independent. When mapping a genotype to a phenotype, we use the same columns of *D* (as defined by 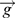) to construct the summed subset in each row. Let's just assume that each trait element is independent which can be stated through the following identity:
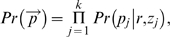
(3)where *k* indexes the phenotypic dimension. Note that the expected value of *z_j_* is *E*(*z_j_*) = *r*/2 and from Equation 2 we get 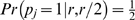. If all rows of *D* possess an equal number of +1's and −1's we find 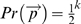 for every phenotype. This generates a uniform distribution of degeneracy levels (and no hidden phenotypes).

Because *z_j_* is sampled from a binomial distribution the number of +1's in each row can diverge from *r*/2, and consequently, as illustrated in Figure 5, bias the probability distribution of phenotypes. Consider the case where several rows of the developmental matrix have *z_j_*>*r*/2. The probability of producing 0's in the phenotype elements that correspond to these rows is very small (note again Equation 2 and the sigmoidal shape in Figure 5). Consequently, producing phenotypes with 0's in all these elements is extremely unlikely (see Equation 3) and these phenotypes are expected to be hidden.

This intuition can also help us to understand the similarity of high frequency phenotypes and the patchiness of the visible phenotype space. Assume that *z_j_*≈*r*/2 only in the first and third rows, and *z_j_*>*r*/2 in all others. Since the phenotypes are biased toward 1's in all elements apart from the first and the third, all phenotypes of the form [–,1,–,1,1,…,1] (where ‘–’ denotes either 0 or 1) are likely to be highly degenerate and will form a dense patch of high frequency phenotypes.

We further confirm this intuition numerically (see Text S1). We applied the mathematical formulation and drew a sample set of *z_j_*'s from a binomial distribution *B*(*r*,0.5). We calculated the probability of obtaining certain phenotypes and showed that the distribution of degeneracy levels is comparable to that obtained with our model. We also demonstrated that the degeneracy levels of neighboring phenotypes are strongly correlated (Text S1).

### Multilayered Developmental Plans

The model we have utilized employs a single layer architecture and might be thought to limit the scope of possible regulatory schemes. Computationally this is the case, as at least two-layers (input layer plus a hidden layer) are required to produce a perceptron that is a universal Turing machine (or universal function approximator), as proven for the Cybenko theorem [22], able to achieve linear separability of inputs as is required by, for example, the XOR function. However, since we show that a single regularity layer compresses the image of the genotypes in the phenotype space, introducing additional layers only produces further canalization and strengthens our findings. We quantify this effect by using an extended multilayered developmental model (see Models) and record the number of unique visible phenotypes and the phenotype distribution obtained after each iteration.

**Figure 6:**
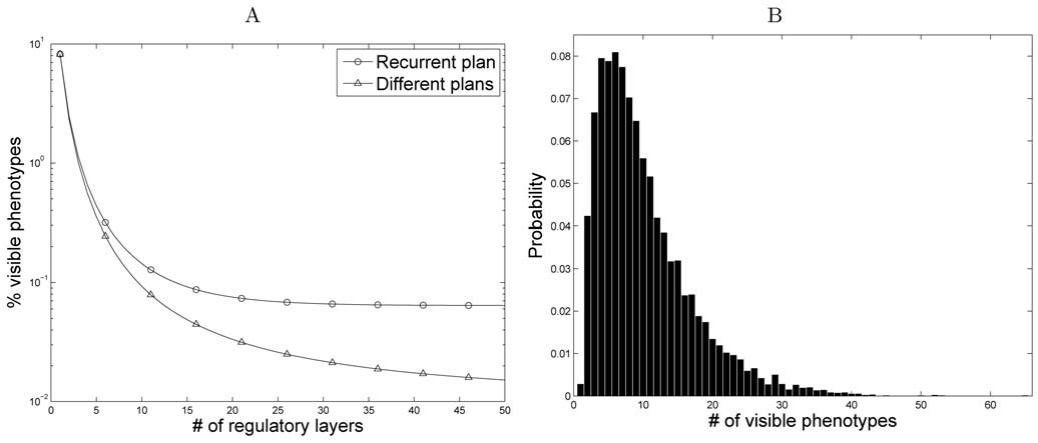
The effect of multilayered developmental plans. (A) The percentage of visible phenotypes out of the potential phenotypes as a function of the number of regulatory layers. The regulatory dimension, *r*, and the phenotypic dimension, *k*, are both set to 14. For a single regulatory layer, the visible phenotypes already constitute only 8.2% of the 2^14^ potential phenotypes, in accordance with our results for the basic model. Introducing additional recurrent layers dramatically decreases the number of visible phenotypes (note the logarithmic scale), reaching 0.06% (approximately 10 phenotypes) with 50 layers. Furthermore, if each regulatory layer incorporates a different developmental plan, the reduction in the number of visible phenotypes as a function of the number of layers is even more extreme. (B) The distribution of the number of unique phenotypes that remain visible when the systems reaches steady state.

First we consider the simple, recurrent, or recursive model, where *D_t_* = *D* for every *t*, and examine the effect of introducing up to 50 regulatory layers. For this recursive scheme, at each additional layer there is a reduction in the number of visible phenotypes and an increase in canalization (Figure 6A). Moreover, the number of visible phenotype reaches an (extremely low) asymptotic value, which is not influences by additional regulatory layers, suggesting that a steady state has been reached.

In order to examine these findings in detail we allow the developmental process to iterate indefinitely until a steady state is reached. This extended model is also strictly comparable with the recurrent models introduced in [15],[17]. As we consider the entire set of possible initial genotypes (rather than a single, predefines, initial phenotype), we apply a slightly more stringent condition for ascertaining the steady state, and require that the set of unique, visible phenotypes does not change (note that this condition also accommodates limit cycle equilibria). Considering 10,000 different developmental plans, we find that on average the number of visible phenotypes at steady state is only 10.3±7 (0.063% of all possible phenotypes), and that this steady state is reached after 17.7±7 layers. Figure 6B further illustrates the distribution for the number of unique visible phenotypes at steady state.

We now examine the behavior of a more general, developmental model, where each regulatory layer can incorporate a different developmental plan. This is closer to natural regulation where we observe multiple layers of post-transcriptional control. As illustrated in Figure 6A, this model yields a more complete reduction in the number of visible phenotypes (as a function of the number of layers). It is enough to note that the ‘all zeros’ phenotype always maps onto itself, and that each layer can map some fraction of the remaining visible phenotypes to this zero-class. This establishes why this model is asymptotically destined to reach a steady state with a single, ‘all zeros’, visible phenotype. This raises an intriguing question as to the optimum number of regulatory layers. Two or more layers offer greater computational power, but at a cost of reduced phenotypic variability.

Finally, we observe a change in the distribution of degeneracy levels as more regulatory layers are introduced. The reduction in the number of visible phenotypes is accompanied by an increase in the probability of highly degenerate phenotypes, and by an overall increase in the extent of degeneracy in the system (Figure S1). This abundance of highly canalized phenotypes further strengthens the conclusions obtained for the original model.

### Developmental Plans with Variable Connectivity Density

The fully connected model analyzed above represents, to some extent, a worst-case scenario in terms of gene interactions, pleiotropy, and epistasis. Here, we examine whether the regulatory bias observed in our model holds when the regulatory plan is less dense, and how the density of the plan influences this bias. There are two competing possibilities to be considered. On the one hand, if the developmental matrix is very parse, there may be entries in the phenotype vector that are never activated. This would further reduce the number of visible phenotypes. On the other hand, for sparse matrices, each phenotypic element is influenced by only a few genes (low epistatsis), making the contribution of each gene to the state of the phenotypic elements higher. Changing one gene in the genotype could change a corresponding element in the phenotype (inducing a steeper gain function - see Figure 3B). In the extreme case of the unit matrix, every change in the genotype induces a change in the phenotype. This would decrease the level of neutrality (degeneracy) of the genotype-phenotype map and consequently produce more visible phenotypes.

**Figure 7:**
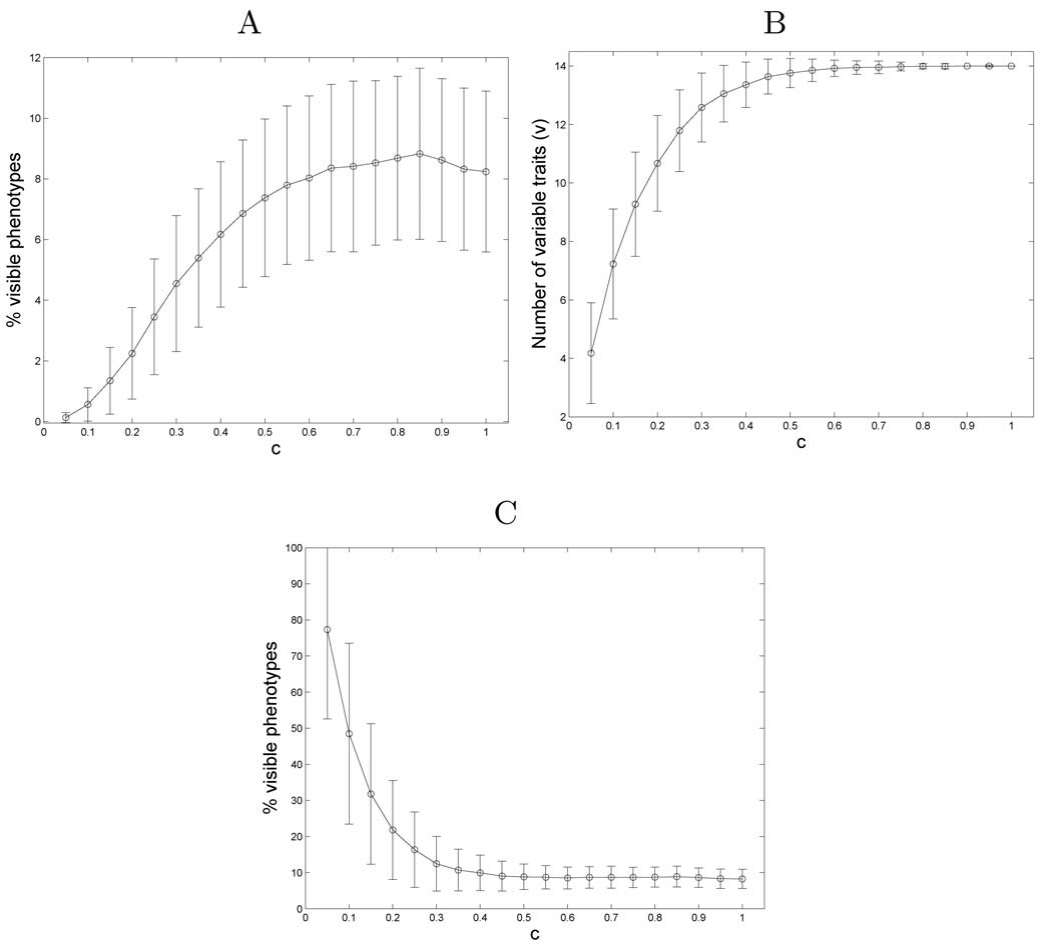
The effect of developmental plan density on phenotype distribution. (A) The percentage of visible phenotypes out of the potential phenotypes as a function of the developmental plan density, *c*. The regulatory dimension, *r*, and the phenotypic dimension, *k*, are both set to 14. Each point represent the average of 1,000 different plans. For a given density value, *c*, each entry in the matrix is attributed with a nonzero value (either +1 or −1) with probability *c*. (B) The number of variable traits, *ν* (i.e., phenotypic elements that are active in at least one phenotype) as a function of the developmental plan density, *c*. The experimental settings are identical to those described in Figure 7A. (C) The percentage of visible phenotypes out of the 2*^ν^* achievable phenotypes as a function of the developmental plan density, *c*.

We find that sparse matrices generate a smaller fraction of visible phenotypes (Figure 7A). For example, in comparison to the 8.2% visible phenotypes obtained for a fully connected plan (*c* = 1), only 3.4% of the phenotypes are visible for a matrix with *c* = 0.25 and only 0.6% are visible for *c* = 0.1 (see Models). It also appears that the maximum number of visible phenotypes (which is still only 8.7% of the total number of potential phenotypes) is produced for an intermediate value of *c*≈0.85. This could be the outcome of a trade-off between the two competing effects discussed above. We note, however, that the influence of an increase in matrix density on the fraction of visible phenotypes diminishes for *c*>0.5.

To disentangle the influence of a sparse matrix density on the fraction of visible phenotypes derived from varying levels of gene interactions (epistatsis), from the effect of reduced phenotypic activation, we control for the number of potentially activated phenotypic elements under each plan. We measure the number of variable phenotypic elements (traits), *ν*, obtained for a range of values of *c* (Figure 7B). A variable trait is defined as a phenotypic elements that can be activated (i.e., assume a value of 1) in at least one of the visible phenotypes produced by a given developmental matrix. As expected, sparser plans result in a lower number of variable traits. If only *ν* traits are variable, the potential number of phenotypes is bounded by 2*^ν^* (rather than 2*^r^*), and it is reasonable to measure the fraction of visible phenotypes in relation to this lower limit. Examining the fraction of visible phenotypes out of the *achievble* (2*^ν^*) phenotypes, the effect of the reduced interaction level is revealed (Figure 7C). Sparser matrices produce a higher fraction of visible phenotypes (reaching almost 80% on average for very sparse plans), owing to the higher marginal contribution each gene makes in determining the state of a phenotypic element. Thus lower epistasis in the sparse matrix allows for a greater per locus contribution to the phenotype.

**Figure 8:**
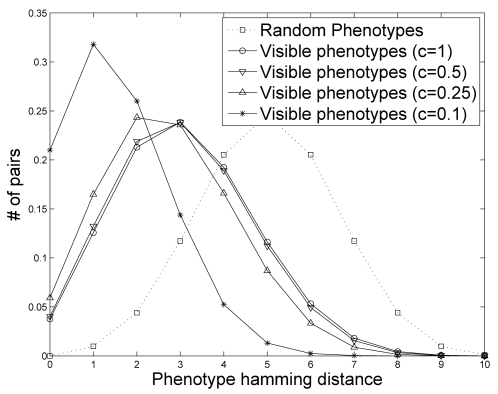
The distribution of pairwise phenotypic Hamming distances among randomly selected phenotypes (not produced by a developmental plan) and visible phenotypes (including all occurrences of each phenotype) produced by developmental plans with varying levels of density, *c*. Each curve represents the average of 100 different plans. Due to computational constraints, the regulatory dimension, *r*, and the phenotypic dimension, *k*, are both set to 10.

We further confirm that the statistical properties of the visible phenotype distribution still holds for sparse matrices. We first compare the distribution of degeneracy levels obtained for varying values of *c* (Figure S2). Although sparse matrices (e.g., *c* = 0.1) produce a more variable distributions, a clear power-law distribution can already be observed for matrices with *c* = 0.25 or higher. The patchiness of the visible phenotypic space is also confirmed for sparse matrices by examining the distribution of pairwise hamming distances among randomly selected phenotypes (Figure 8). Sparse matrices induce an even more pronounced patchy phenotypic space, largely as a result of a reduction in the number of visible phenotypes these plans produce (see Figure 7A).

In summary, we find that reducing the level of regulatory interactions in the developmental plan produces two competing effects. The first effect is to reduce the number of visible phenotypes and increase the patchiness of the visible phenotypic space. These are the result of an increase in inactivation for a number of phenotypic elements. The second effect is an increase in the number of visible phenotypes. This is a result of the higher marginal contribution of each gene to determining the state of each associated phenotypic element. Both these effects are prominent for sparse matrices, but become negligible for density values in the range *c*>0.5. This might suggest that the phenotypic bias generated by the fully connected matrix remains relevant for typical, empirically derived networks, for which density values below or close to 0.5 have been observed [23].

### An Ontogenetic-Phylogenetic Model

**Figure 9:**
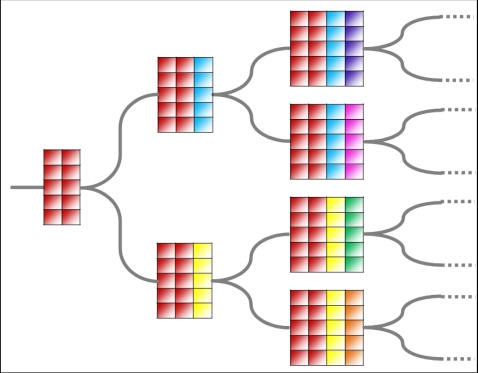
Simulating the evolutionary process forward through time. Similar colors denote shared regulatory wiring.

**Figure 10:**
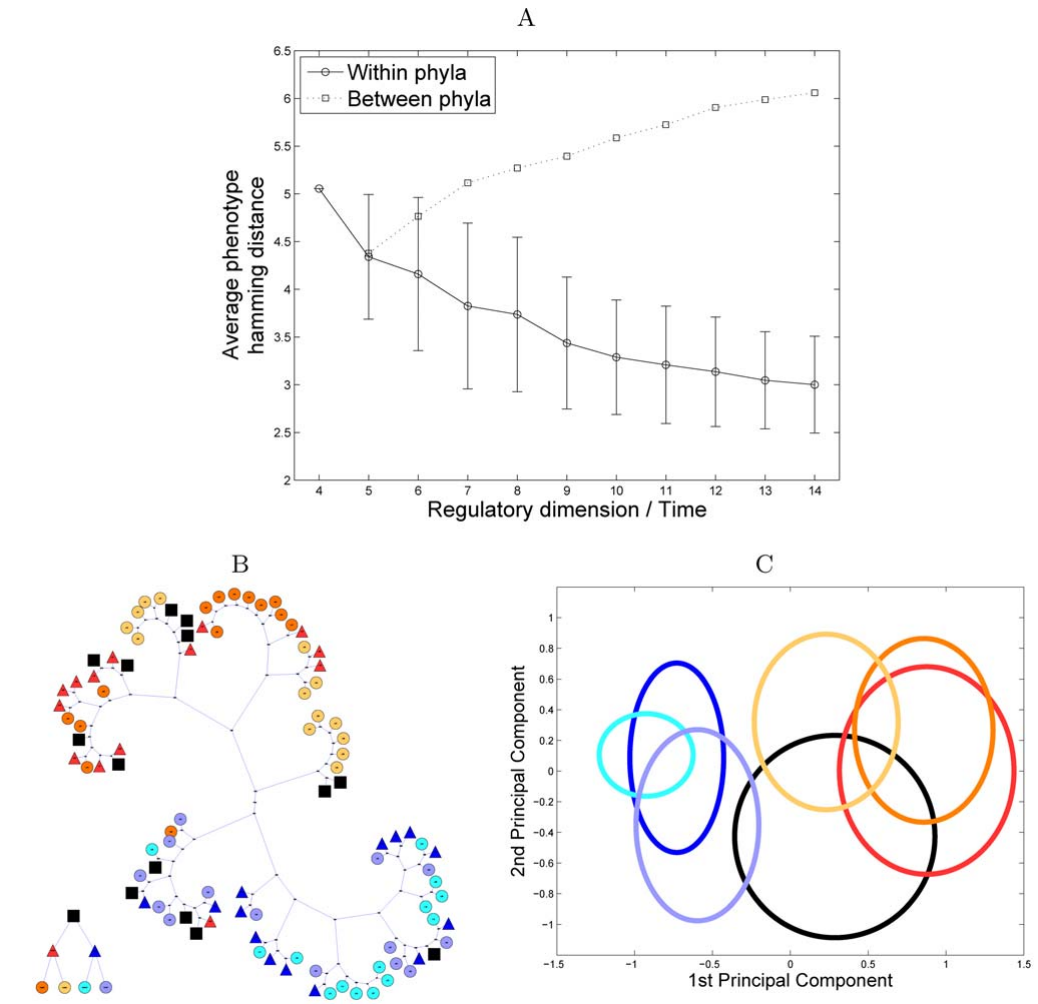
Phenotype distribution in an ontogenetic-phylogenetic model. (A) The average pairwise Hamming distance between visible phenotypes within and between phyla. Each phylum corresponds to a developmental plan, and the set of the most frequent visible phenotypes produced by this plan represent species. The ancestral phyla is employing a developmental plan with *r* = 4 and *k* = 14. In each branching event, each of the two descendant phyla add an additional regulatory element with random connectivities preserving the ancestral component of the developmental plan (Figure 9). This branching process continues until we get the 1024 most recent phyla, each employing a developmental plan with *r* = 14 and *k* = 14. (B) A phylogenetic tree including phenotypes from derived and ancestral phyla. The tree is reconstructed by computing the pairwise Hamming distance matrix between all phenotypes and applying a neighbor-joining algorithms. Rectangular, triangular, and circular nodes represent phenotypes from the ancestral phylum, intermediate phyla, and derived phyla respectively. Phyla within each phylogenetic level are illustrated with different colors. The small tree on the bottom left corner illustrates the phylogenetic tree of different developmental plans (using the same color coding as that used in the main tree). Phenotypes (or ‘species’) of different phyla differ only in the developmental plan and not in genotype, but the resulting tree successfully clusters the members of each phyla. Furthermore, the members of intermediate phyla are correctly clustered, spanning the same phylogenetic space as their descendants. Members of the ancestral phylum (represented by black rectangles) span similar regions to those covered by all derived phenotypes. (C) Representation of ancestral, intermediate, and derived phenotypes according to the first two principle components. Ellipses illustrate the mean and variance for each phylum. The color coding is identical to that used in the phylogenetic tree.

Finally, we consider the effects of the developmental map on phylogenetic regularities. Since we are focusing on the evolution of development bearing on phenotypic diversity and disparity, we do not consider the evolution of the structural genes, but only regulatory interactions. We assume in the following treatment that developmental plans evolve incrementally and neutrally by addition of new genetic regulatory elements into existing regulatory networks. Consider, for example, an ancestral developmental plan that possesses *r_a_* transcription factors, controlling *k* target genes. Descendant developmental plans acquire *r_b_*>*r_a_* transcription factors (still controlling the same *k* genes), where all descendant plans share an identical regulatory wiring for the ancestral *r_a_* transcription factors, and differ in the wiring of the derived factors (Figure 9). Following findings in the previous section, we focus only on the most frequent phenotypes produced by each plan as evolutionarily representative of the complete, visible phenotype set. By focusing on the most frequent phenotypes, we are considering those phenotypes most likely to be observed. We are interested in the phylogenetic distribution of phenotypes generated by the evolutionary sequence of developmental plans. We observe that the phenotypes comprising a single developmental plan, become more similar throughout the evolutionary process, whereas disparity among members of different plans increases (Figure 10A). This process relates to an increase in the regulatory dimension of the genome, and hence illustrates how regulatory evolution promotes increasing phyletic disparity while decreasing phenotypic disparity.

To illustrate the similarities and relationships among phenotypes, specifically between current phenotypes and ancestral phenotypes, we perform a phylogenetic analysis. We follow the evolutionary process described above (see also Figure 9), starting with an ancestral group that embodies a developmental plan with *r* = 4 and *k* = 14. A first branching event results in two intermediate groups, each with *r* = 9 and *k* = 14. A second branching event results in four groups, each with *r* = 14 and *k* = 14. We consider a collection of phenotypes comprising the most frequent visible phenotypes in the most derived groups, the intermediate groups, and the ancestral group, and reconstruct a phylogenetic tree relating these phenotypes (Figure 10B). This tree is exact as we preserve the complete evolutionary history of each lineage. The resulting tree not only clusters the derived groups correctly, but also demonstrates that intermediate and ancestral groups span the same phenotypic space as their descendants. Note, in particular, that phenotypes in the ancestral group cover (though, more sparsely) most of the space covered by the derived groups. A similar pattern can be observed by means of a principal components analysis of the phenotypic set (Figure 10C).

## Discussion

The implications of developmental dynamics for evolutionary dynamics has become an area of outstanding interest as details of the networks underlying body plans have been elucidated [6],[24]. There is a growing interest in the stability of phenotypes [25], mechanisms facilitating and constraining the development and plasticity of traits [26], and the implications of development on both micro and macro-evolutionary trends [7], [9]–[11].

In this paper we present a schematic model of development based on a plan resembling a cis-regulatory architecture [14],[15], where transcription factors bind to promoters leading to the expression or inhibition of downstream, structural genes. The parsimonious structure of this model is able to reproduce important empirical regularities in the evolution of development, allowing us to exclude the need to construct unnecessarily complicated hypothesis. We find that regulatory mechanisms promote genetic epistasis in gene expression, leading a large fraction of phenotypic space to become concealed. This dramatically limits the number of available phenotypes. This finding suggests that the sparseness of morphological varieties in nature [27] can be at least partially attributed to the constraining properties of genetic networks, particularly those networks regulating the activity of downstream targets of activators. This property of an abstract regulatory process has been discussed by Gould, when he writes that, “phenotypic' similarities *arise instead as a constraint based on common genesis from a source that imposes limitations or sets preferred channels of change from within*” [28]. This interpretation of convergence is to be distinguished from any reduction in phenotypic variation subsequent to development arising through stabilizing selection acting against the deleterious effects of perturbations of complex regulatory networks.

This is in the statistical sense, a null model for development, ignoring important properties of dynamics, pattern formation and selective feedback. All of these processes play a significant role in the formation of the phenotype and yet all of them are neglected. This follows from the assumption that a powerful null model seeks to account for a large percentage of variation with a minimum of functional assumptions. Hence the rather abstract character of the model, and its inability to predict particular, empirical details of development.

### Degenerate Maps and Morphological Grades

The distribution of phenotypic degeneracy levels recalls results from the genotype/phenotype map induced by RNA secondary structure [20], where it has been shown that frequencies of planar structures are highly nonuniform (following a generalized form of Zipf's law) resulting in few common structures and many rare ones. There are two important differences between simple genotype/phenotype maps and our results. First, whereas the RNA genotype/phenotype map is the outcome of physical interactions between base pairs, the mapping presented in this paper is the result of a developmental scheme, representing interactions among multiple transcripts. Second, for RNA secondary structure, the space of potential shapes is considerably smaller than the sequence space. RNA studies focus on the distribution of visible phenotypes and on the organization of the visible phenotypic neutral networks. We consider the size and structure of the space *not* covered by neutral networks.

The molecular study of developmental maps in multicellular lineages has tended to focus on changes over a small number of generations, typified by studies of homeotic mutants. Paleontologists have become interested in the macroevolutionary implications of developmental evolution, in particular, the production of features associated with higher taxonomic levels [11]. The benchmark example of what we might call ‘developmental macroevolution’ is the Cambrian radiation associated with a rapid proliferation of highly disparate, multicellular animals [12]. The putative causes of this radiation include the accumulation of atmospheric oxygen [10], a snowball earth scenario [29], as well as a variety of putative developmental innovations including the emergence of *Hox* cluster of genes [6], and the co-opting of regulatory networks for new structures and functions [30].

Whatever factors might have lead to the original ‘explosion’ of varieties, we are able to show with a suitable model for development, that simple, low dimensional ancestral regulatory networks will tend to produce a higher disparity among the set of most frequent phenotypes than is the case for, derived, high-dimensional networks. This is because the ancestral programs are less constrained by regulatory epistasis. Moreover, developmental evolution generates anisotropic phenotypic variation, towards an increasingly clustered occupancy of phenotypic subspaces. These results agree with prior studies showing a tendency towards a clustering of phenotypes and a deceleration of diversification in abstract morphospaces that arise through branching random walks [13] at levels above individuals, or through random rates of speciation and extinction imposed on a background rate of discrete anagenesis [31].

It has been suggested that developmental plans constitute a mechanical explanation and justification for phylogenetic grades [7]. These results support this hypothesis, as each developmental plan represents a conserved core responsible for imposing a shared pattern of expression on a lineage of organisms. Critically, these organisms can share the bulk of their genes and yet remains significantly different when these genes are expressed through their unique developmental programs. It remains to be determined why these programs remain relatively uniform through time. One possibility is that changes to these programs are more deleterious than changes to the non-regulatory quotient of the genome [7]. Another possibility, is that since selection acts only indirectly on the genetic program but directly on the traits that it generates, the selective pressure on the plan is weak, and when coupled to the canalizing effects of the plan, severely decelerates the evolutionary process. In an important sense, it is this property of variation in structural genes compared to invariance of the developmental plan that allows for the emergence of high level grades. If this constraint is relaxed, phenotypes are more uniformly distributed, making the concept of, for example, phyla an arbitrarily placed epiphenomenon of phylogenetic trees.

### Developmental Macroevolution

The role of development in generating, or constraining, biotic diversity has been one of the most active debates in evolutionary biology [32]–[34]. The roots of this debate go back to the study of homologies and questions over physico-chemical verses genetically-selected rules of growth. One merit of simple developmental models is to illustrate how these two positions reflect necessary, complementary properties of generic developmental programs. Regulatory epistasis introduces non-linearities into development, allowing similar genotypes to generate significant divergence among phenotypes, whereas degeneracy tends to contract the occupancy of morphospace and bias phenotypic samples. Of great interest is how these structural properties of development have themselves been modified over the course of evolutionary time, potentially changing the tempo and mode of the evolutionary process. One of the paradoxical implications of this study has been to show how innovations in development (arising through increasing regulatory dimensions) that lead to an increase in the volume of accessible phenotypes, can lead to a reduction in selective variance (through increasing regulatory epistasis), so whereas the potential for novel phenotypes increases, the fraction of space these phenotypes occupies tends to contract. Hence the evolutionary process moves from a macro-configuration, sampling distant regions of space sparsely, to a micro configuration, sampling local regions of space at high resolution. This is analogous to an annealing process, whereby as an optimization process proceeds, the solutions become more frequent and more densely localized around the putative solution points.

## Supporting Information

Figure S1 A loglog plot of the distribution of degeneracy levels among visible phenotypes using varying number of regulatory levels. The settings are identical to those described in Figure 3A in the main text, but using (A) 1, (B) 2, (C) 5, (D) 10, (E) 25, and (F) 50 regulatory layers. Each point denotes the expected number of distinct phenotypes with a certain degeneracy level and is an average over 10,000 different plans. Evidently, introducing additional regulatory layers further increases the extent of canalization, producing an increasing number of highly degenerated phenotypes. These plots are generated using the same recurrent developmental plan in each level (as in [1],[2]), but using different plans produces qualitatively identical results.
(0.81 MB TIF)

Figure S2 A loglog plot of the distribution of degeneracy levels among visible phenotypes for varying regulatory densities. The settings are again identical to those described in Figure 3A in the main text, but with the matrix density, c, set to (A) 0∶1, (B) 0∶25, (C) 0∶5, and (D) 1. Each point denotes the expected number of distinct phenotypes with a certain degeneracy level and is an average over 1,000 different plans. It appears that the power-law distribution of degeneracy level is showing already in relatively sparse matrix (e.g., only 25% nonzero entries).
(0.66 MB TIF)

Figure S3 (A) A loglog plot of the distribution of degeneracy levels among visible phenotypes as obtained by the numerical analysis. Each point denotes the expected number of developmental plans in which the ‘half ones’ phenotype obtains a certain degeneracy level, and is averaged over 1,000,000 different plans. From symmetry considerations, this distribution reflects the expected distribution of degeneracy levels among all visible phenotypes in a randomly generated developmental plan. Note that the point associated with degeneracy level 0 (i.e., a hidden phenotype) is not included. (B) The degeneracy level of the ‘almost half ones’ phenotype, as a function of the degeneracy level of the ‘half ones’ phenotype in the same plan, demonstrating the high correlation between the degeneracy levels of neighboring phenotypes. For convenience, we draw the points associated with only 1,000 plans.
(0.60 MB TIF)

Text S1 Supporting Text: Numerical Analysis
(1.27 MB PDF)
