# Supplementary figures and images for: An End to Endless Forms: Epistasis, Phenotype Distribution Bias, and Nonuniform Evolution
Source: PLoS Comput Biol. 2008 Oct 24;4(10):e1000202. doi: 10.1371/journal.pcbi.1000202 (PMC2562988; doi:10.1371/journal.pcbi.1000202)

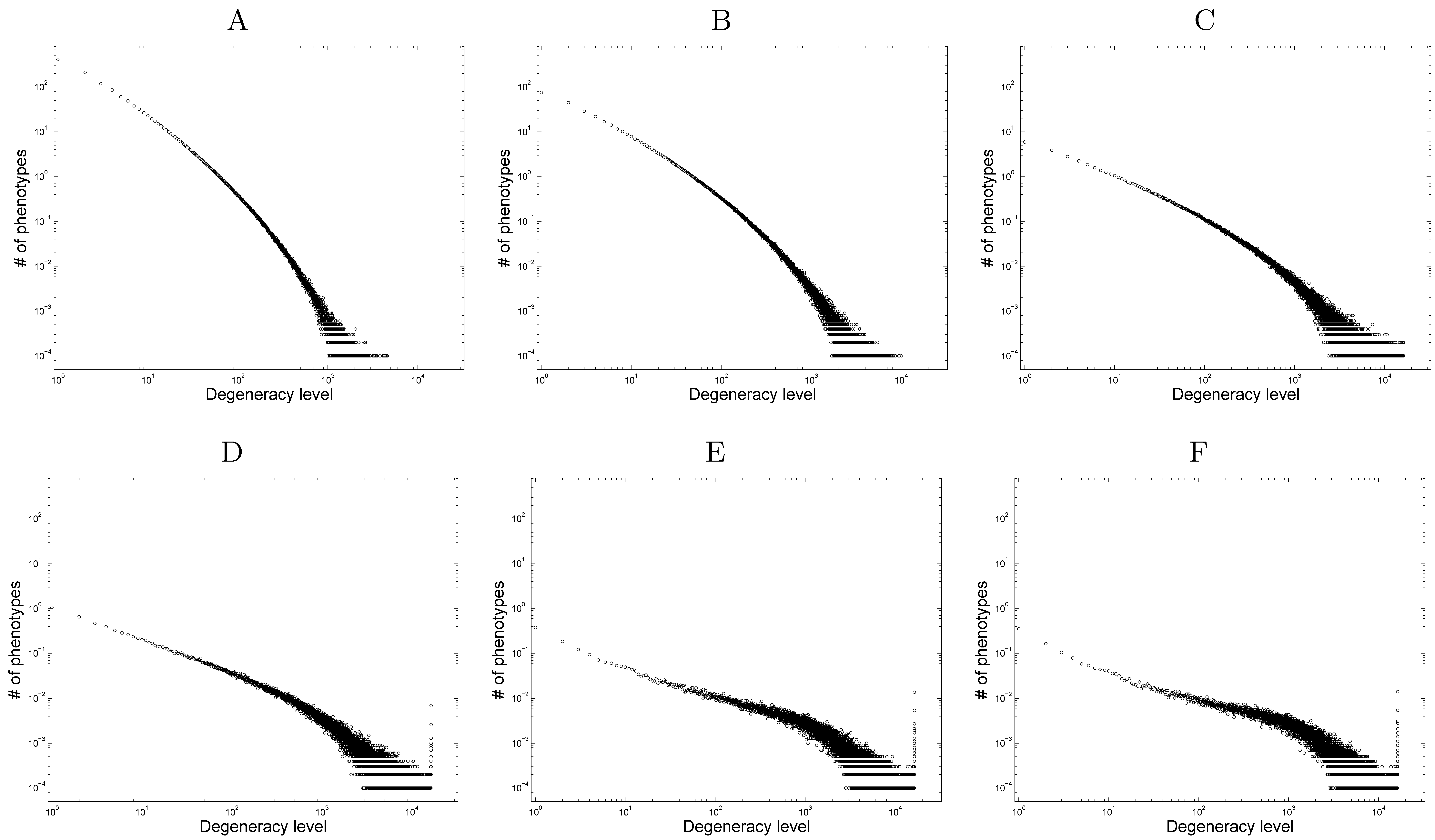

Supplement: Figure S1 — A loglog plot of the distribution of degeneracy levels among visible phenotypes using varying number of regulatory levels. The settings are identical to those described in Figure 3A in the main text, but using (A) 1, (B) 2, (C) 5, (D) 10, (E) 25, and (F) 50 regulatory layers. Each point denotes the expected number of distinct phenotypes with a certain degeneracy level and is an average over 10,000 different plans. Evidently, introducing additional regulatory layers further increases the extent of canalization, producing an increasing number of highly degenerated phenotypes. These plots are generated using the same recurrent developmental plan in each level (as in [1],[2]), but using different plans produces qualitatively identical results. (0.81 MB TIF) [file pcbi.1000202.s001.tif]

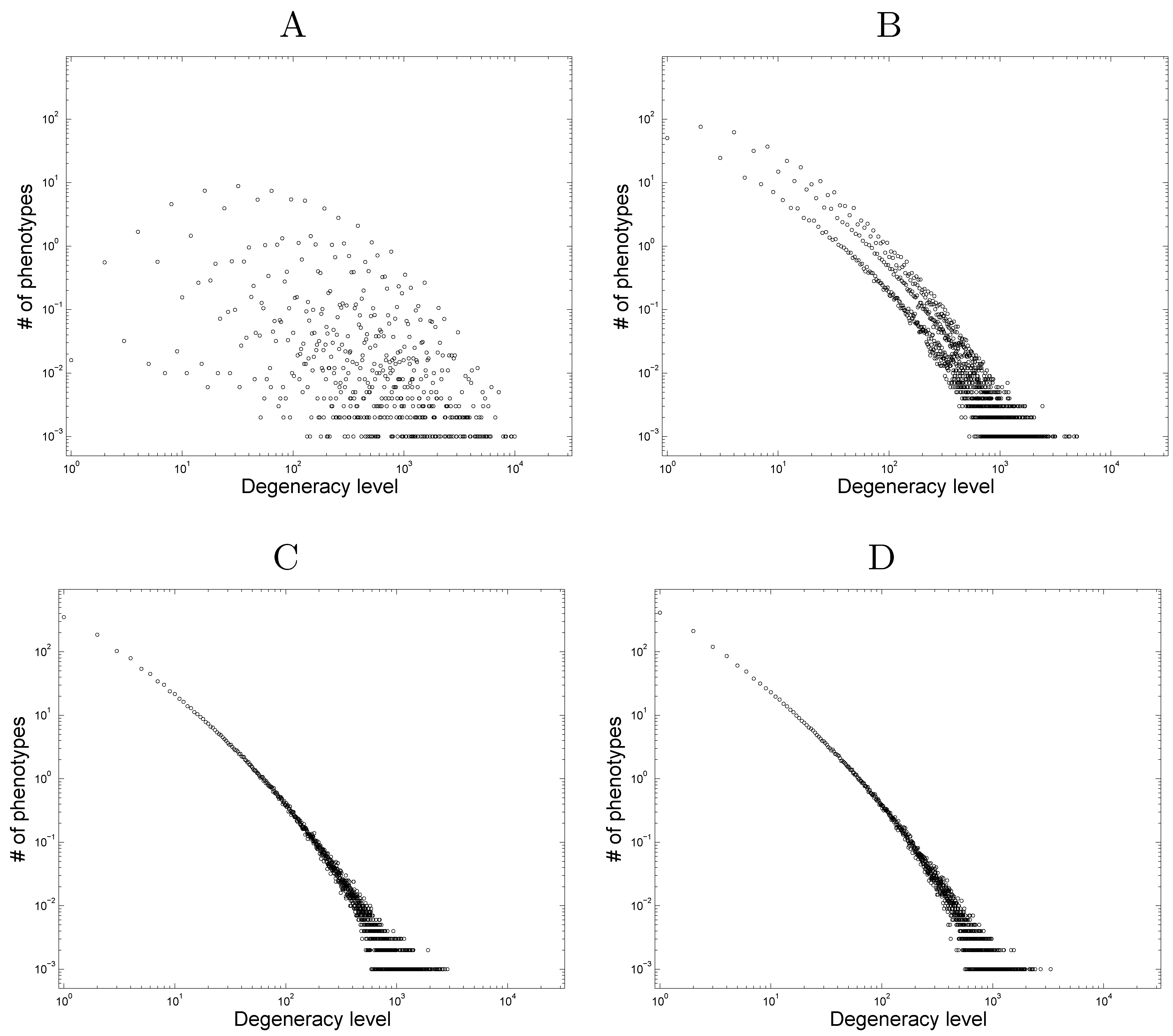

Supplement: Figure S2 — A loglog plot of the distribution of degeneracy levels among visible phenotypes for varying regulatory densities. The settings are again identical to those described in Figure 3A in the main text, but with the matrix density, c, set to (A) 0∶1, (B) 0∶25, (C) 0∶5, and (D) 1. Each point denotes the expected number of distinct phenotypes with a certain degeneracy level and is an average over 1,000 different plans. It appears that the power-law distribution of degeneracy level is showing already in relatively sparse matrix (e.g., only 25% nonzero entries). (0.66 MB TIF) [file pcbi.1000202.s002.tif]

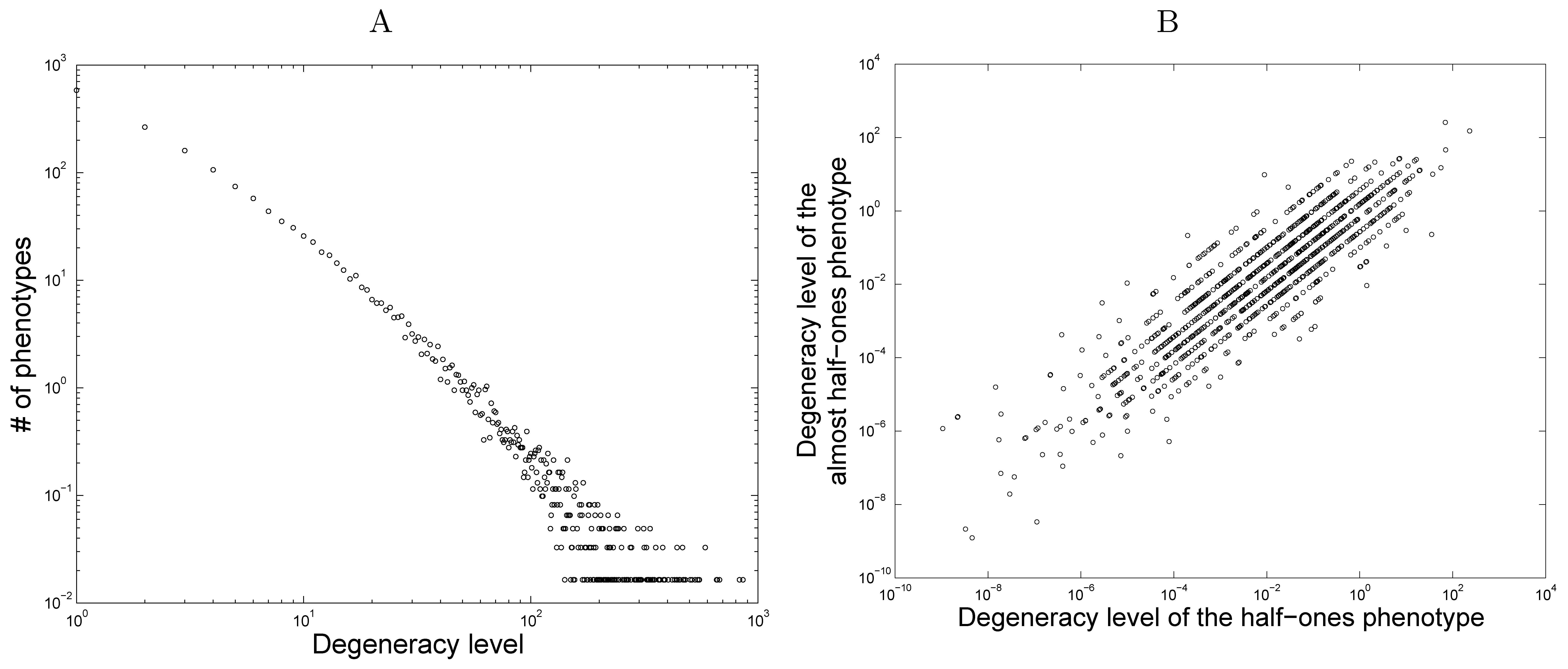

Supplement: Figure S3 — (A) A loglog plot of the distribution of degeneracy levels among visible phenotypes as obtained by the numerical analysis. Each point denotes the expected number of developmental plans in which the ‘half ones’ phenotype obtains a certain degeneracy level, and is averaged over 1,000,000 different plans. From symmetry considerations, this distribution reflects the expected distribution of degeneracy levels among all visible phenotypes in a randomly generated developmental plan. Note that the point associated with degeneracy level 0 (i.e., a hidden phenotype) is not included. (B) The degeneracy level of the ‘almost half ones’ phenotype, as a function of the degeneracy level of the ‘half ones’ phenotype in the same plan, demonstrating the high correlation between the degeneracy levels of neighboring phenotypes. For convenience, we draw the points associated with only 1,000 plans. (0.60 MB TIF) [file pcbi.1000202.s003.tif]
