# Supplementary material for: An End to Endless Forms: Epistasis, Phenotype Distribution Bias, and Nonuniform Evolution
Source: PLoS Comput Biol. 2008 Oct 24;4(10):e1000202. doi: 10.1371/journal.pcbi.1000202 (PMC2562988; doi:10.1371/journal.pcbi.1000202)
Supplement: Text S1 — Supporting Text: Numerical Analysis (1.27 MB PDF) [file pcbi.1000202.s004.pdf]

An End to Endless Forms: Epistasis, Phenotype Distribution Bias, and Non-Uniform Evolution

Elhanan Borenstein, David C. Krakauer

Supporting Information

Supporting Text

Numerical Analysis

This mathematical analysis presented in the main text and the intuition we provided for the characteristic features of the phenotype distribution generated by our model can be confirmed numerically. We draw sets of z_j's ($j = 1, 2, \ldots, k$) from a binomial distribution $B(r, 0.5)$. Each set represents a randomly generated developmental plan (reduced only to the z_j's statistics). We use Eqs (1),(2), and (3) in the main text to calculate the probability of obtaining the 'half ones' phenotype from a random genotype in each plan. The 'half ones' phenotype is defined as a phenotype having 1's in the first $k/2$ elements and 0's in the rest. We approximate the degeneracy level of the 'half ones' phenotype by multiplying this probability by the total number of genotypes (r^2). When this predicted degeneracy level is smaller than 1, we declare that the 'half ones' phenotype is hidden under this plan. From symmetry considerations (and ignoring dependencies between the degeneracy levels of the different phenotypes), the probability distribution for the 'half ones' phenotype across different developmental plans (i.e., different sets of s_j's) provides a good approximation for the expected distribution of degeneracy levels of all phenotypes in a

single, randomly generated plan.

Figure S3A illustrates the distribution of degeneracy levels obtained through this analysis. The distribution is very similar to the distribution obtained for the full model (see Figure 3A in the main text). The predicted percentage of hidden phenotypes (i.e., those for which the calculated degeneracy level is smaller than 1) is 90%, a slightly smaller value than that obtained for the full model simulation ($\approx$ 92%). The discrepancies are likely to be the outcome of various dependencies ignored in the simplified analysis. We have also calculated for each set of z_j's the predicted degeneracy level of the 'hamming half ones' phenotype, a phenotype which is one point mutation away from the 'half ones' phenotype. As illustrated in Figure S3B, the predicted degeneracy levels for these two neighboring phenotypes are strongly correlated ($r = 0.87$ $p < 10^{-300}$, Spearman correlation test).

Supporting Figures

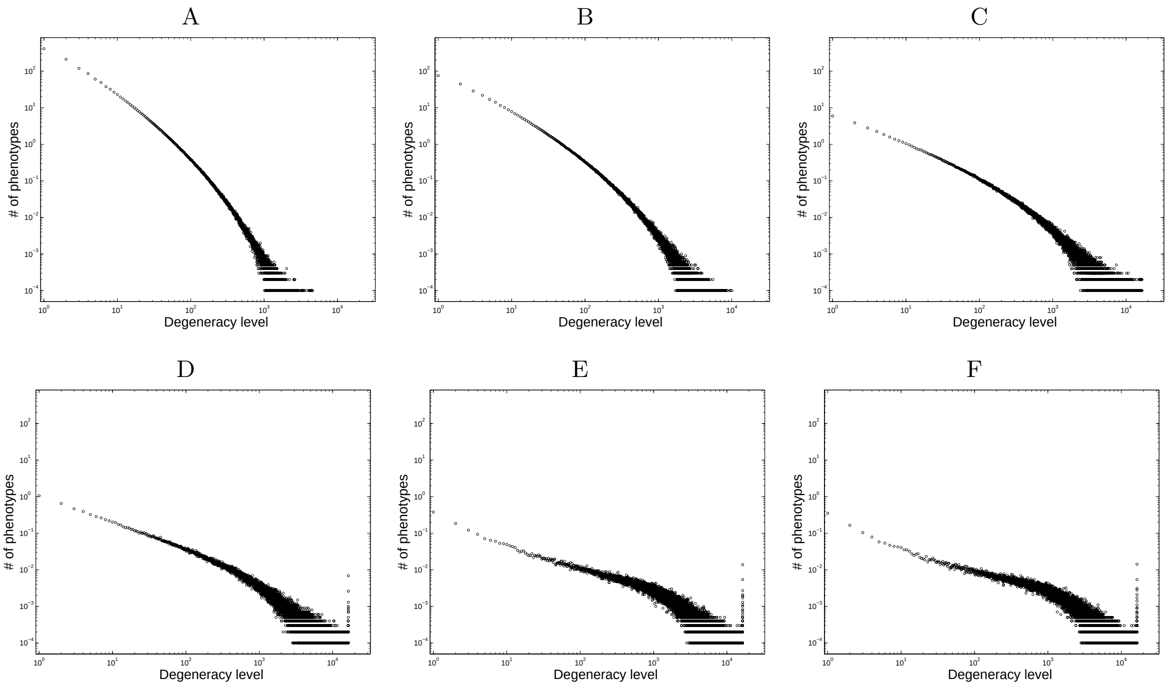

Figure S1: A loglog plot of the distribution of degeneracy levels among visible phenotypes using varying number of regulatory levels. The settings are identical to those described in Figure 3A in the main text, but using **(A)** 1, **(B)** 2, **(C)** 5, **(D)** 10, **(E)** 25, and **(F)** 50 regulatory layers. Each point denotes the expected number of distinct phenotypes with a certain degeneracy level and is an average over 10,000 different plans. Evidently, introducing additional regulatory layers further increases the extent of canalization, producing an increasing number of highly degenerated phenotypes. These plots are generated using the same recurrent developmental plan in each level (as in [1, 2]), but using different plans produces qualitatively identical results.

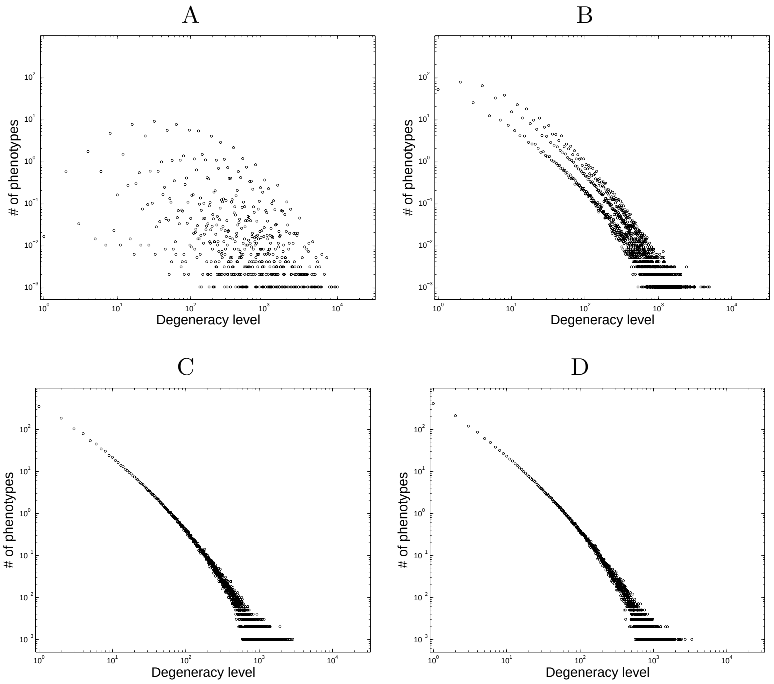

Figure S2: A loglog plot of the distribution of degeneracy levels among visible phenotypes for varying regulatory densities. The settings are again identical to those described in Figure 3A in the main text, but with the matrix density, c, set to **(A)** 0.1, **(B)** 0.25, **(C)** 0.5, and **(D)** 1. Each point denotes the expected number of distinct phenotypes with a certain degeneracy level and is an average over 1,000 different plans. It appears that the power-law distribution of degeneracy level is showing already in relatively sparse matrix (e.g., only 25% nonzero entries).

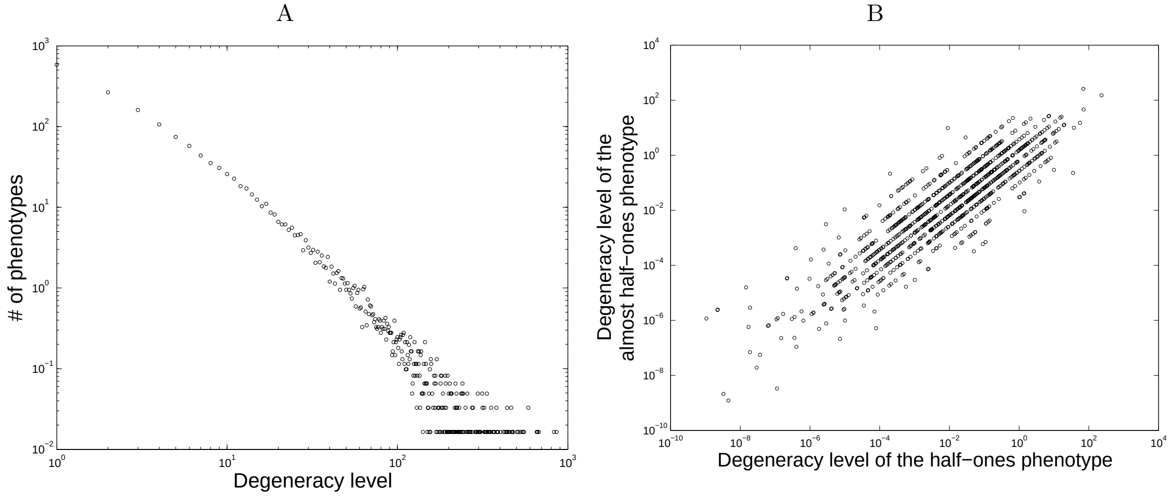

Figure S3: **(A)** A loglog plot of the distribution of degeneracy levels among visible phenotypes as obtained by the numerical analysis. Each point denotes the expected number of developmental plans in which the 'half ones' phenotype obtains a certain degeneracy level, and is averaged over 1,000,000 different plans. From symmetry considerations, this distribution reflects the expected distribution of degeneracy levels among all visible phenotypes in a randomly generated developmental plan. Note that the point associated with degeneracy level 0 (i.e., a hidden phenotype) is not included. **(B)** The degeneracy level of the 'almost half ones' phenotype, as a function of the degeneracy level of the 'half ones' phenotype in the same plan, demonstrating the high correlation between the degeneracy levels of neighboring phenotypes. For convenience, we draw the points associated with only $1,000$ plans.

References

[1] Wagner A (1996) Does evolutionary plasticity evolve? Evolution 50:1008–1023.

[2] Siegal M, Bergman A (2002) Waddington's canalization revisited: Developmental stability and evolution. Proceedings of the National Academy of Sciences 99:10528–10532.
